# Behavioral, cognitive, emotional and social engagement in mathematics learning during COVID-19 pandemic

**DOI:** 10.1371/journal.pone.0278052

**Published:** 2022-11-22

**Authors:** Dirgha Raj Joshi, Krishna Prasad Adhikari, Bishnu Khanal, Jiban Khadka, Shashidhar Belbase

**Affiliations:** 1 Mahendra Ratna Campus Tahachal, Tribhuvan University, Kirtipur, Nepal; 2 Nepal Open University, Lalitpur, Nepal; 3 Department of Mathematics Education, Central Campus, Tribhuvan University, Kirtipur, Nepal; 4 Faculty of Social Science and Education, Nepal Open University, Lalitpur, Nepal; 5 Department of Curriculum and Instruction, College of Education, United Arab Emirates University, Al Ain, Abu Dhabi, United Arab Emirates (UAE); University of Eastern Finland: Ita-Suomen yliopisto, FINLAND

## Abstract

A meaningful engagement of learners is critical in the quality teaching and learning of mathematics at school level. Learner engagement has been an ongoing issue in mathematics classrooms in Nepal and elsewhere. In this context, this study aimed to examine the level of engagement (behavioral, social, emotional, and cognitive activities) and their association with learning mathematics through the virtual mode of instruction during the pandemic. The cross-sectional online survey design was employed among 402 secondary-level mathematics teachers in Nepal. Descriptive statistics, correlation, and structural equation modeling were the major statistical techniques used in research. The findings indicate that the level of behavioral, social, emotional, and cognitive engagement of students was found to be high in the online mode of instruction. Additionally, cognitive engagement has significant highest impact on social, behavior, and emotional engagement.

## Introduction

Learners’ engagement is a significant factor in enhancing their interest and attentiveness toward learning by decreasing their frustrations [1, 2]. In this context, learner engagement in mathematical tasks and activities may enhance achievement by impacting students’ emotional, cognitive, and behavioral development [3, 4]. The difficult situation created by the COVID-19 pandemic had an impact on parents’ feelings and students’ ways of life [5, 6]. That situation eventually impacts the engagement of students in the learning process. Engagement is an important construct in mathematics learning [7] that connects theoretical and practical problems in cognitive engagement and students’ access to knowledge and information to solve mathematical problems [1]. In the same vein, affective and behavioral engagement focus on students’ involvement in mathematical tasks and activities [8] are also crucial.

Collaborative learning and the interaction of learners may have a favorable impact on their critical thinking [9]. Learner engagement remained a central aspect of teaching and learning mathematics throughout history with many reforms (e.g., New Math, Back to Basics, and Standard Movement). This construct has been a major point of interest to educational stakeholders in terms of quality assurance in the online mode of instruction, especially during the COVID-19 pandemic. It also focuses on the psychological commitment of the students to stay in the learning process for the acquisition or construction of knowledge with creativity and critical thinking [10]. When physical classes were closed during the COVID-19 pandemic, quality online education was critical to ensure that the learners were effectively and adequately engaged in the learning process [11, 12]. It was necessary to maintain learner engagement in the learning process [13] with active engagement by thinking, talking, and working with the content, teachers, and peers [11, 14–16]. Without an active engagement of learners, the whole pedagogical action would not be effective [17].

The literature shows that that there are effects of different engagements on learning. However the current research is focused on the effect of each type of engagements on the other and student learning. On the other hand, the situation of social, emotional, behavioral, and cognitive engagement in mathematics learning through online during the COVID-19 pandemic in a developing country, such as Nepal, is the main issue raised in the research. Research on the engagement pattern of students in the online mode of delivery is quite a novel issue in Nepal where it is an important component of learning. So, this study focused on finding the answers to the following questions:

What is the status of learners’ engagement (behavioral, social, emotional, and cognitive) in learning mathematics during the COVID-19 pandemic in Nepal?

How do the behavioral, social, emotional, and cognitive engagements of learners in mathematics learning predict to each other?

The institutions of Nepal did not have any preparation or planning for a virtual mode of instruction during the COVID-19 pandemic. However, some of the institutions in the urban areas of the country adopted a virtual mode of instruction for continuing their annual instructional activities. The main concern of this study was how the learners were engaged in mathematics learning in their behavioral, social, emotional, and cognitive activities. This study examined the status of learners’ engagement in mathematics learning during the COVID-19 pandemic. Furthermore, the study focused on how the behavioral, social, emotional, and cognitive engagement of students predict each other. The findings of the study are significant for policy makers, trainers, and other stakeholders in identifying the engagement of learners in the virtual mode of instruction and their association with designing ideas and programs for enhancing different engagement-related activities of mathematics learners. This study is limited to a developing country context like Nepal considering the COVID-19 pandemic and mathematics as one of the compulsory subjects in school education. Hence, the findings of this research can also be applicable to a similar crisis in the future. The study is further grounded on the following three scenarios that depict the context of this study.

A mathematics teacher enters a high school classroom. All the students stand up from their seats and greet their teacher, and the teacher greets them back. Then he asks the students to take their seats. He asks them how they are doing. He asks them whether all of them are fine. He asks all the students to observe a short silence in their seats with their eyes closed. He asks them to take a long breath. After this moment of silence for about a minute, he reminds the students of the prior lessons and tasks to bring some home-made learning materials. The students (both male and female) are sitting on the desks and benches arranged in rows with minimal space for movement. The teacher tries to contextualize the topic probability with the ongoing world cup football match (held in 2014). He then relates the concept of probability to the possibility of students’ being passed or failing on the tenth-grade national examination. He continues asking students questions about the probability of getting a head or a tail when tossing a coin. He talks about a sample space in probability by tossing a dice. He writes the sample space on the white board by tossing a dice. This linear fashion of question asking by the teacher and students answering these questions in a chorus (all or many at a time) continues throughout the class [18].

In another class, a mathematics teacher enters a classroom. All the students stand up from their seats and greet him. He also greets them back. He asks them how they are doing. The students reply to him that they are doing well. They ask him how he is doing. He replies that he is also fine. He then asks them to take their seats. All the students sit on their benches. He reminds them of an assignment from the day before on the topic of height and distance (in trigonometry). He asks them if all the students have done it and brought their work to the class. In a chorus, students say that they have brought their work to the class. He also tells them that there is no problem with the assignment. He re-iterates if they have any confusion, and the students state that they don’t have any confusion about it. The teacher points at a student on the front row and asks him what the task was. The student explains that it was the construction of a clinometer. He asks a group of students (named "group D") to show their clinometer to the class. Then, he asks all the students if they have their clinometer with them in the class. All the groups say that they have it with them. Some students show their clinometer to the teacher. He reminds them that the objective of the lesson is to use the clinometer to measure the height of an object. He mentions that they are going to find the height of the school building by using a clinometer. He further states that they are going to measure the height of a tree in the school compound. He points out a tree outside the classroom through the window. He tells them that they will also measure the height of a nearby stupa. He writes these objectives on a whiteboard at the front of the class. He divides all the students into four groups. He assigns them the task of measuring the height of different objects (e.g., a tree, a school ceiling, and a stupa). He also demonstrates how to use their clinometers to measure the angle of elevation from a distance at the top of an object. Then, the students in each group go to measure the height of the objects assigned to them. Once the students gather data from their measurement, the teacher discusses with the class how they measured the height of objects. He brings all the students back to the class. He asks all the group leaders to explain how they measured the height of objects by using angles of elevation (for a tree and a stupa) or angles of depression (for the height of a school building from the roof top) [19].

In the third classroom context, a mathematics teacher does not show up in a classroom. The school has been closed due to the COVID-19 pandemic. The teacher, who has access and resources to make classroom videos, records his lecture and uploads it to YouTube. The teacher greets the students in grade 6 and welcomes them to his virtual lesson. He then introduces the unit and topic of discussion as sets, elements of sets, and writing a set and its elements in set notations. He reminds the students that they have experienced the concept of sets in everyday life. He shows a bundle of marker pens and explains that it is a set of marker pens. Then, he shows a set of geometrical instruments in a box. He shows students (audiences) a protractor, a ruler, a compass, a setsquare, and a divider from the box. He explains that these individual pieces form elements of a set of geometric instruments. He then demonstrates pictures of some fruits and vegetables on the white board and asks the students if the elements can be well defined. He explains whether it could be defined as a set or not. He then separates two sets: a set of vegetables and another set of fruits. He continues by explaining the elements of sets and how to write these elements in mathematical notation. This online class is a lecture without students joining at the same time. In this sense, it is an asynchronous class where students are not directly engaged in the learning process at the same time when the teacher is giving his lecture. That means there is a lack of student engagement in the teaching-learning process. It is a one-way lecture by the teacher to the intended learners [20].

These scenarios represent more or less most of the mathematics classrooms in Nepal before the COVID-19 pandemic (scenario 1 and 2) and during the pandemic (scenario 3). When we look at these classes from the point of view of learner engagement, they are mostly active listeners. They listen to the teachers most of the time, with some opportunities to answer the questions asked by the teacher. Their answers, in most cases, do not reflect creativity, critical thinking, and mathematical reasoning. Rather, these answers are short and chorus (as a group) responses with a few opportunities to think and solve problems. They may conduct some experiments but do not contribute much in their mathematical thinking because these experimental contexts are mostly repetitions of the same concept in action. Therefore, learner engagement in mathematics is not very constructive and active in terms of the construction of meaning of concepts, procedures, and applications of mathematics. This was an even more serious issue when the schools were closed due to the COVID-19 pandemic in 2020 and 2021. Many schools tried to engage their students’ learning through alternative methods, for example, online videos and synchronous meetings with Zoom and Microsoft Teams applications. Learner engagement was severely disrupted during the pandemic. In this context, the present study examines learners’ engagement in terms of behavioral, cognitive, social, and emotional engagement during the COVID-19 pandemic in Nepal.

### Literature on learner engagement

Learner engagement in online mode is significantly positively correlated with motivation, satisfaction, and overall performance [21–23]. Additionally, it may reduce the attrition rates of students [24]. Learners’ engagement can promote instructor-student and peer-to-peer interactions, prompting feedback in a respectful and active learning environment [25]. Nonetheless, it is a challenge to maintain the same level of engagement in online and virtual classes despite the fact that there are technologies (e.g., applications, software, and programs) with the capacity to create engaging learning environments [26]. The learners’ engagement in such technological environment may depend on the availability, accessibility, and usability of the tools in the learning activities [27].

Moreover, a study by the Education Review Office [ERO] has shown that school-level mathematics achievement in Nepal is comparatively lower than in other subjects [28, 29]. ERO [29] has suggested providing maximum learning opportunities for students. Similarly, Cevikbas and Kaiser [30] state that a low level of engagement of the student in mathematics learning can be a factor for the low level of achievement. Learner engagement patterns in the virtual learning environment are relatively novel issues in the context of school mathematics in Nepal. This issue needs to be explored in order to examine how the learner engagement was affected by the COVID-19 pandemic, especially when the schools and educational institutions had to rely on virtual classes.

Although the pandemic situation has played a significant role in transforming towards the virtual mode of instruction [31], the level of preparation for online education was almost none at the beginning when the government of Nepal announced the first lockdown on March 24, 2020. When the country had to go through a prolonged closure of schools and universities, then the government of Nepal formed different committees and strategic plans to deal with educational losses due to the lockdowns and closures. Some of these plans include COVID-19 Education Cluster Contingency Plan 2020, Alternative Learning Facilitation Guidelines, Emergency Action Plan for School Education, School Reopening Framework, and Closed User Group (CUG) Service Implementation Guidelines [31] to continue educational activities from school to university levels. After a period of uncertainty and chaos in education due to sudden disruption and lack of preparation, the schools and universities slowly moved toward distance learning from radio and television, online teaching, home-teacher support programs, community-based tutorial classes, sharing digital hangouts, and sharing digital resources as the major instructional practices during the pandemic in Nepal [32]. Despite these efforts to resume education, access to the Internet, digital devices, and resources were found to be poor in rural areas of Nepal [33]. Instructional activities at the university level were transformed to an online mode. However, all the students still had a shortage of basic infrastructure and resources, especially learners from the remote areas, financially poor family backgrounds [34], infrastructure for virtual system of learning, skillful human resources, virtual instructional practices [35], self-motivation [36], and technical support [37] which were necessary components for the effective virtual learning.

The virtual learning environment differs from the physical in terms of tutors’ and learners’ presence, teaching-learning activities, use of learning materials, and assessments. Moreover, a study conducted by the Education Review Office [ERO] has shown that school-level mathematics achievement in Nepal is comparatively lower than in other subjects, such as language and science [28, 29]. ERO [29] has suggested providing maximum learning opportunities for students and Cevikbas and Kaiser [30] stated low level of engagement as an important factor for the low level of achievement, which are necessary to further explore to enhance the existing mathematics achievement. In this context, a further study required to explore the effectiveness of learners’ engagement in learning during the lockdown and closure of physical classes due to the COVID-19 pandemic. Considering the specific context of mathematics learning in a virtual class, this study has explored the learners’ engagement in mathematics learning in the virtual mode of instruction during the COVID-19 pandemic. There is some literature (published or unpublished) about students’ engagement in the physical setting of the classroom in the Nepalese context, but a study on the engagement pattern of students in the virtual learning environment is a relatively novel issue for Nepal.

The education sector of Nepal has been affected by the COVID-19 pandemic for the last three years since the outbreak of the coronavirus. All educational activities in the country were entirely in face-to-face mode before the pandemic at the school level. During the lockdown, several educational institutions of Nepal had been practicing online and virtual pedagogy without any pre-planned strategies. Although the pandemic situation has played a significant role in transforming towards the virtual mode of instruction [31], the level of preparation for online education was almost none in many schools and higher education institutions, although some institutions had online and virtual classes, for example, the Distance Learning Center of Tribhuvan University and the School of Education, Kathmandu University.

Teachers can enhance learners’ engagement in face-to-face education by meeting with individual students in a physical context by keeping a careful eye on pupils’ actions and recognizing a student’s psychosocial behavior [38]. Although physical meeting with pupils was difficult in the online format, the contents and related activities in online environment were all geared toward students’ participation in the learning process with engaging instructional approaches with social-emotional support from their teachers, parents, and classmates [39]. Literature shows that learner engagement is a combination of attention and commitment of students at five levels: rebellion, retreatism, ritual compliance, strategic compliance, and authentic engagement [40]. In this context, teacher evaluations of pupils, observation of student performances, self-reports, and biosignal measurement are important to monitor the level of engagement [41]. The facial expressions can be used to evaluate student participation in terms of emotions such as grief, rage, disgust, fear, surprise, and delight [41]. In face-to-face interaction, peer or group facilitation can be very effective. However, teacher facilitation seems more successful than peer facilitation in online learning [42]. Teacher questioning and commenting on students’ tasks and activities may have a positive effect on leaners’ motivation and performance [16] through social, pedagogical, and technical aspects of engagement [43].

### Theoretical framework

**Fig 1:**
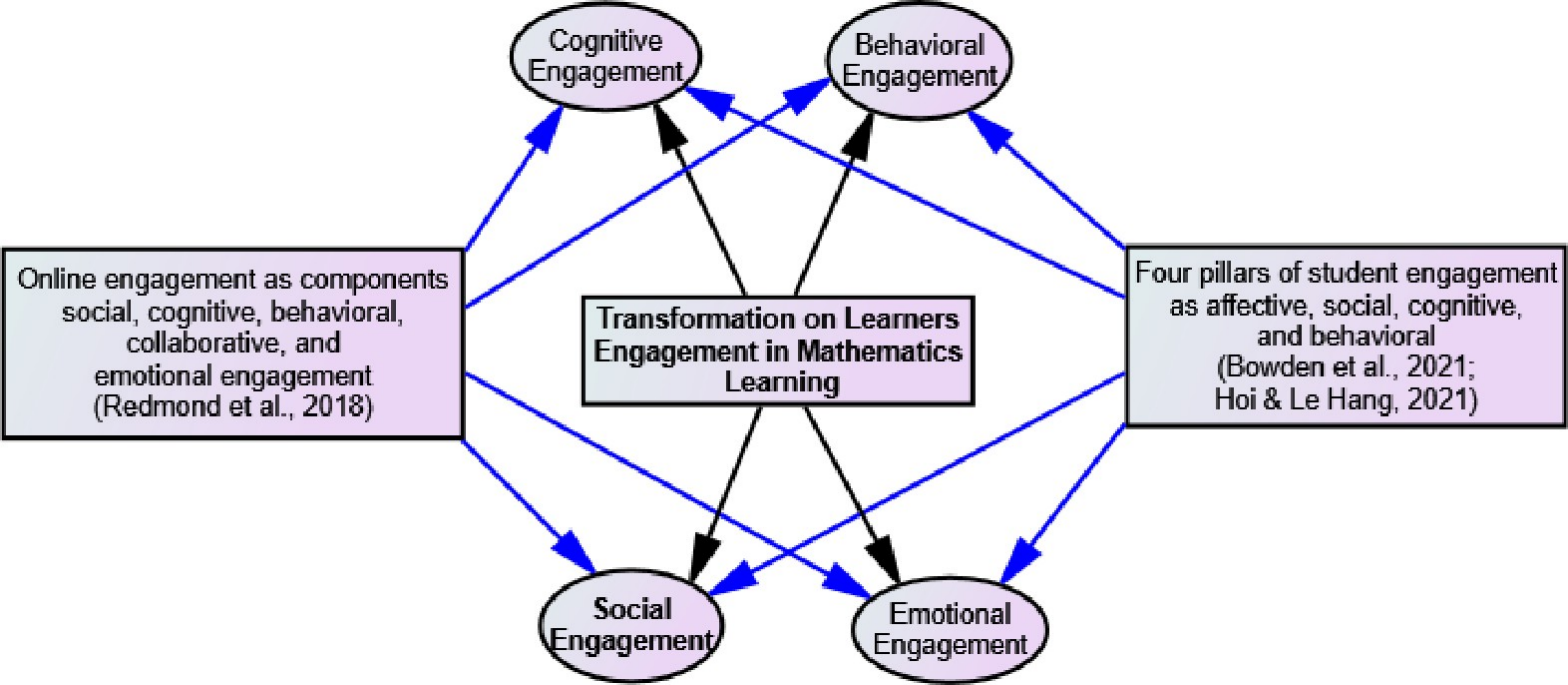
Theoretical framework of learner engagement in terms of social, behavioral, cognitive and emotional engagement (black arrow represents the domain of research and blue arrow represents the support connection of theories).

There are various competing and contrasting ideas about students’ engagement in an online environment [44]. For example, Bowden et al. [45] proposed four pillars of student engagement: affective, social, cognitive, and behavioral (Fig 1). In the same line, Redmond et al. [46] suggested five components of online engagement: social, cognitive, behavioral, collaborative, and emotional engagement. Redmond et al. [46] emphasized collaborative engagement for peer or group learning in a sense similar to social engagement [45]. We developed a study tool based on the ideas of [45] and a bi-factor exploratory structural equation-model by Hoi and Hang [44].

*Affective engagement* relates to the emotional experiences of students [47], which are manifested through enjoyment, pride, and satisfaction as positive, and anger, anxiety, and frustration as negative states [48]. Both student interest and enjoyment may produce positive emotions by increasing engagement [49], which are tightly related to their learning, achievement, and satisfaction [50]. *Social engagement* may create positive bonds between peers and instructors [50], reducing the risk of isolation, disconnection, and dropouts [44]. Cooperation, listening to others, punctuality in class, and maintaining balanced relations with instructor and peers are the components of social engagement in the context of the classroom [50]. Outside of the classroom context, social engagement is based on the shared values, interests, or purposes of participation in a community clubs, study groups, and student organizations [51, 52]. Students in an online learning mode need to feel that they are not alone in their learning but connected to a group of learners [10]. *Behavioral engagement* is associated with academic performance and participatory actions and activities [47, 53], which may include participation in class discussions, involvement in curricular and co-curricular activities, time spent on academic work, perseverance, and resiliency [38]. The participatory behavior of students in co-curricular activities can influence behavioral engagement [54]. *Cognitive engagement* is related to an internal psychological process [55], which also refers to a strategic learning approach that promotes self-regulated deep learning strategies [8, 56], with higher-order thinking skills [3], with frequent and interactive engagement [57].

Based on these theoretical constructs, the researcher developed new constructs which explains the effect of each domain of learners’ engagement as behavior, emotional, social, and cognitive engagement to others by assuming each domain as dependent variable ones.

The theoretical framework helped us to construct four hypotheses as:

H1: There is a significant positive effect of emotional, social, and cognitive engagement on behavioral engagement.
H2: There is a significant positive effect of behavioral, cognitive, and emotional engagement on social engagement.
H3: There is a significant positive effect of social, behavioral, and emotional engagement on cognitive engagement.
H4: There is a significant positive effect of social, behavioral, and cognitive engagement on emotional engagement.

## Methodology

### Study setting

The study was carried out among the mathematics teachers in Nepal at school level. The research focused on the online mode of instruction of mathematics which was recently introduced in Nepal during the COVID pandemic. Hence, only digitally trained teachers were considered for the study sample. The research was entirely online survey-based design with a cross-sectional online quantitative study. There are not any ethical boards for research in Nepal to review research proposals and tools. However, an approval to this study was obtained from the Department of ICT Education, Central Campus Kirtipur, Tribhuvan University, Nepal for its authenticity and ethical concerns.

### Sample and sampling technique

High school mathematics teachers were contacted through professional organizations and institutions such as the Nepal Mathematical Society and the Council of Mathematics Education during professional development activities and training in virtual mode. A total of 1,333 mathematics teachers were sent the online questionnaire through a link in the Google Form. The questionnaire included an introductory paragraph informing participants about the purpose of the study, time expected to fill up the questionnaire, their right to withdraw from the study, potential risk, data use, and protection of their personal identity. If they consented, then they would be able to go to the next page with the questions/items. If they did not consent, then they would exit from the survey. This way, the participants chose to participate in the study voluntarily. Out of the 1,333 high school teachers who received the online questionnaire, only 402 (30.16%) responded and participated in the survey. By taking a 5% confidence interval in the assumed population, the appropriate sample size was 298 [58]. However, the respondent of this study was 402. Hence, the simple of the research was representative and sufficient so that the results could be generalizable to the entire population of high school teachers in Nepal.

### Participants information

The participants were not limited based on any socio-demographic characteristics like gender, teaching level, qualification, and years of teaching experience. However, out of 402 participants 9.2% were female and 90.8% were male. Based on the experience of teachers, 14.4% have less than 5 years of experience, 31.1% have 5–10 years, and 54.5% have more than 10 years of teaching experience. Similarly, 20.9% teachers were from the basic level (teaching at grades 1–8) and 79.1% of them were from the secondary level (teaching at grades 9–12). Additionally, 3.2% of the teachers had intermediate (equivalent to high school), 20.6% of them had bachelor’s degrees, 72.9% of them had master’s degrees, and 3.2% of them had an MPhil or PhD qualification.

### Description of variables with hypothesized models

**Fig 2:**
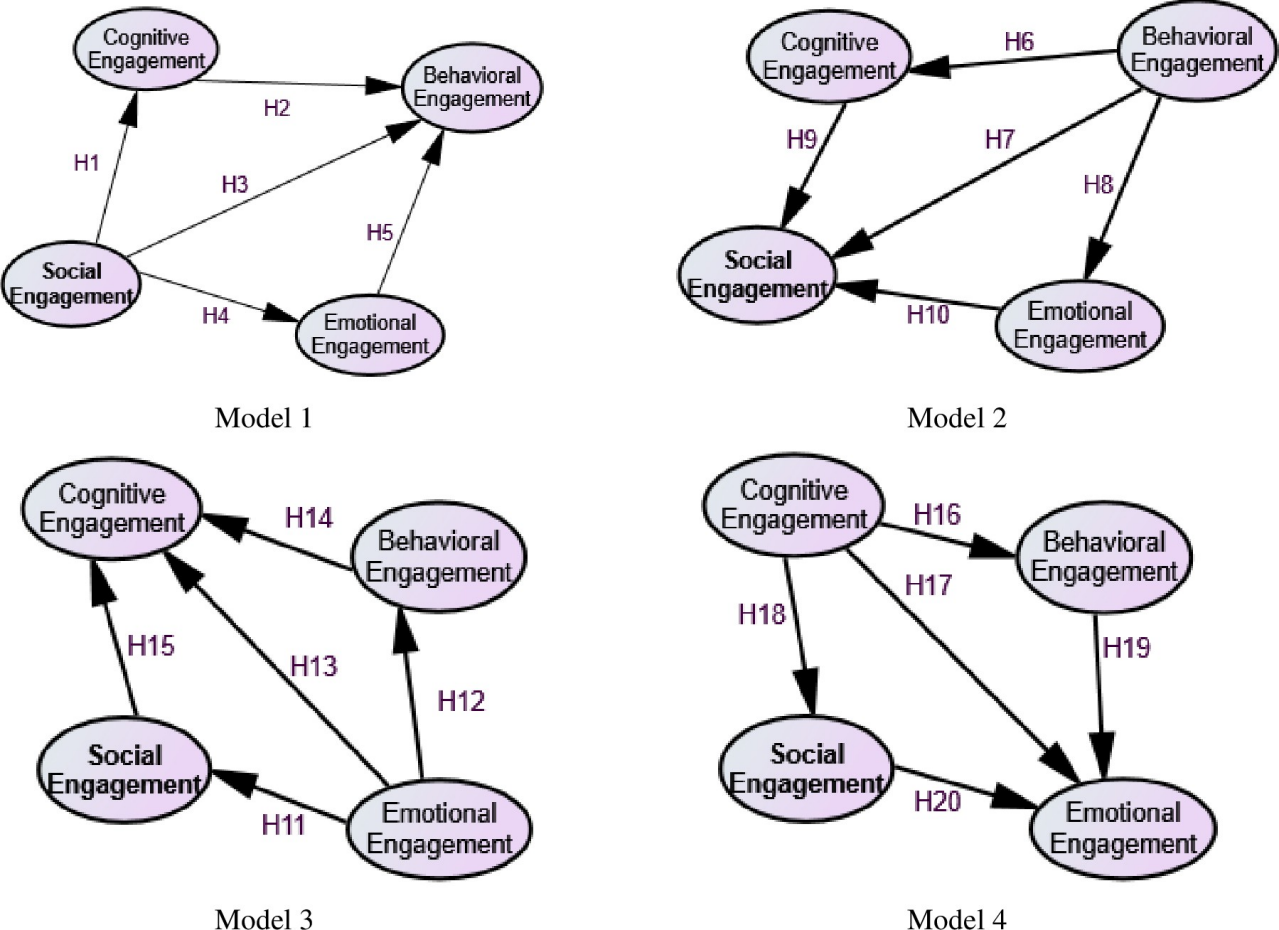
Conceptual framework (Hypothesized models).

The four hypotheses formulated were used to develop a conceptual model interrelating items and the engagements. Each of these models with relevant items have been discussed under separate sub-headings and Fig 2. The related items in this model are in S1 Appendix.

### Behavioral Engagement (BE)

Behavioral engagement included five items concerning participants’ academic activities and transformation on their learning habit [47, 52] through technological tools in the online learning mode (e.g., students are more active to accomplish the homework and assignment on time). In the analysis, the contribution of each item to behavioral, emotional, social, and cognitive engagement were measured. Furthermore, the hypothesized Model 1 was developed to assess the effect of emotional, social, and cognitive engagement on behavioral engagement.

### Social Engagement (SE)

Social engagement consisted of four items related to social activities of students through the use of technology, like observing community norms, participation in social activities, formation and participation in online forums or study groups, and ways of working in groups through the use of technology [49]. A hypothesized Model 2 was used to examine the effects of behavioral, social, emotional, and cognitive engagements on social engagement.

### Cognitive Engagement (CE)

The cognitive engagement consisted of five items like enhancement of learner’s motivation and encouragement towards managing self-learning materials [54], develop or transform students as self-directed learners [8, 55] (Wu et al., 2021), and collaborate with peers in assignments during the online learning [56]. A hypothesized Model 3 was use to examine the effects of behavioral, social, and emotional engagement on cognitive engagement.

### Emotional Engagement (EE)

Support of technology in transforming the interests and feelings of students, reducing their boredoms, increasing happiness, feeling comfortable participating in online discussion, and reducing anxieties were measured under the emotional engagement [48]. A hypothesized Model 4 was developed to examine the effect of behavioral, social, and cognitive engagement in emotional engagement.

### Reliability and validity

**Fig 3:**
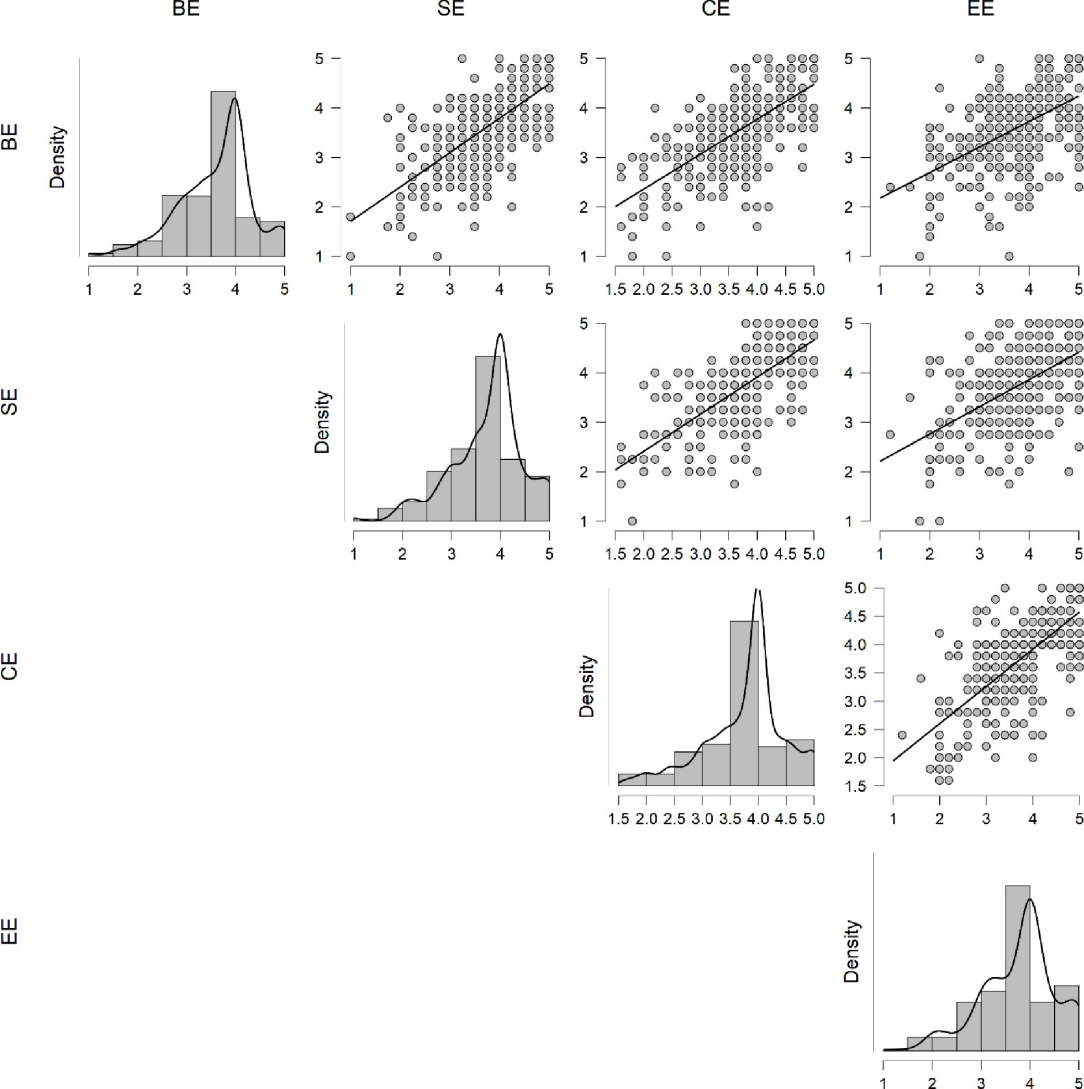
Correlation plot based on the four dimensions.

The confirmatory factor analysis (CFA) was calculated to estimate the factors of the engagement-related items. The model consists of four factors: behavioral, cognitive, social, and emotional engagement. The standardized factor loadings were found to be 0.65 to 0.98 (Fig 3), and the item reliabilities were found to be 0.85 to 0.99, indicating that the items seemed good indicators for the latent factors. The validity and reliability of the instrument were ensured by using different statistical techniques. The reliability was calculated by Cronbach alpha and overall reliability was found to be 0.94, whereas the dimension wise reliabilities were 0.87 for behavioral engagement, 0.85 for social engagement, 0.88 for cognitive engagement, and 0.87 for emotional engagement. The internal reliability coefficient of Cronbach’s alpha greater than 0.70 deemed acceptable [59, 60]. Face, discriminant, and construct validity were measured to establish the validity of the instrument. For content validity, the instrument was shared with education and mathematics education-related six experts and their feedback were incorporated before piloting by modifying some items. For the discriminant validity, square root of average variance extracted (AVE) with the correlation value between the latent variables based on four dimensions separately [61, 62]. However, the correlation coefficient was found to be high in some cases which showed a problem in the reliability [63]. Hence, heterotrait–monotrait (HTMT) ratio correlation technique [64] was performed. The HTMT values were found to be less than the threshold criteria (<0.85), which ensured the discriminant validity [65]. The AVE between all four dimensions was found to be 0.55 to 0.69 (Table 1), which exceeded the threshold criteria of 0.50 [62]. Hence the construct validity was established.

**Table 1:** Detail of reliability and validity.

Factors	Cronbach’s Alpha	CR	AVE	HTMT Analysis			
				SE	BE	CE	EE
SE	0.86	0.77	0.61				
BE	0.87	0.86	0.55	0.80			
CE	0.88	0.88	0.61	0.84	0.78		
EE	0.87	0.92	0.69	0.66	0.60	0.49	

### Data analysis techniques

Descriptive statistics and SEM were the major statistical techniques applied in the research. The measures of mean and standard deviation (SD) were applied to show the item and dimension-wise status of transformation on learners’ behavioral, cognitive, emotional, and social engagement during the COVID-19 pandemic in online learning of mathematics. The levels of items and dimension-wise engagements were determined based on the mean scores. The mean score of 3.67–5 was considered high, mean score of 2.34–3.66 as moderate, and mean less than 2.34 was considered low. The one-sample t-tests were performed for the significance of the values of items was based on the assumed population mean of 3. The structural equation modeling (SEM) was used to measure the effects of social, cognitive, behavioral, and emotional engagement on each other. Before using the SEM, the assumptions related to an outlier, VIF, tolerance, and model fit indices were examined. The JASP application was used for the calculation of descriptive results and the correlation diagrams, whereas AMOS-23 was used to calculate the results of SEM.

## Results

Table 2 shows that among the four dimensions of engagement, the level of cognitive (Mean = 3.77, SD = 0.71), emotional engagement (Mean = 3.77, SD = 0.75), and social engagement (Mean = 3.74, SD = 0.73) of students in the online mode of teaching were found to be high (Mean>3.67 whereas behavioral engagement (Mean = 3.61, SD = 0.74) was moderate. Level of learners’ engagement on engaging for self-learning (Mean = 3.78, SD = 0.90) and responsible for their learning by doing (Mean = 3.72, SD = 0.89) were found to be high whereas that levels were found to be moderate in remaining variables as active to accomplish the homework and assignment, aware on self-evaluation, and practiced collaboration with peers on academic discussion under behavioral engagement. However, the student who could engage in self-learning had the highest mean score (Mean = 3.78, SD = 0.90), and students who were more active to accomplish homework and assignment had the lowest mean score (Mean = 3.43, SD = 0.96) as compared to item-wise results under behavioral engagement. All items, such as “students can participate in different social activities through technology”, “teachers can promote students’ social engagement by balancing structure with student autonomy”, and “students can learn ways of working in a group through technology” had a high level of engagement except one item as “students can observe community norms by using technology under social engagement.” In the item, “students can learn ways of working in a group through technology under the social engagement dimension” had the highest mean score (Mean = 3.80, SD = = 0.86) and the item “students can observe community norms by using technology” had lowest mean score (Mean = 3.61, SD = 0.88).

**Table 2:** Result of items and dimension wise descriptive statistics with reliability and validity (n = 402).

Items with categories	Mean	SD	t-value
**Behavioral Engagement (BE)**	3.61	0.74	16.50*
The student seemed engaged in self-learning (BE1)	3.78	0.90	17.21*
Students are more active to accomplish the homework and assignment on time (BE2)	3.43	0.96	8.95*
Students are becoming more aware of self-evaluation (BE3)	3.53	0.95	11.18*
Students have practiced collaboration with peers on academic discussion (BE4)	3.59	0.88	13.40*
Students are responsible and focused during the online classes (BE5)	3.72	0.89	16.24*
**Social Engagement (SE)**	3.74	0.73	20.19*
Students can observe community norms by using technology (SE1)	3.61	0.88	13.89*
Students participate in different social activities through technology (SE2)	3.78	0.92	17.02*
Students are active in forming a study group in the online forum and take participate in group work (SE3)	3.77	0.87	17.70*
Students can learn ways of working in a group through technology (SE4)	3.80	0.86	18.68*
**Cognitive Engagement** (CE)	3.77	0.71	21.71*
Online learning enhances learner’s motivation towards learning (CE1)	3.85	0.86	19.74*
Online pedagogy has encouraged learners to manage self-learning materials (CE2)	3.76	0.85	18.05*
Technology can develop/transform students as self-directed learner (CE3)	3.75	0.91	16.53*
Students learned to give and receive peer feedback and correct their own mistakes (CE4)	3.72	0.86	16.86*
Students can collaborate with peers in doing homework and assignment (CE5)	3.76	0.85	17.90*
**Emotional Engagement** (EE)	3.77	0.75	20.73*
Technology can transform the interest and feelings of students (EE1)	3.91	0.85	21.33*
Technology can reduce boredom (EE2)	3.72	0.91	15.88*
Technology can increase happiness (EE3)	3.85	0.88	19.29*
Students feel comfortable participating in online discussion (EE4)	3.74	0.95	15.66*
Technology can reduce anxiety (EE5)	3.64	0.99	13.03*

Under the Cognitive engagement, the levels of engagement were found to be high in all items as “online pedagogy can enhance learners’ motivation”, “online pedagogy has encouraged learners to manage self-learning materials”, technology can develop/transform students as self-directed learners, students learned to give and receive peer-“feedback and correct their own mistakes”, and “students can collaborate with peers in doing homework and assignment.” Within this dimension, the item “online pedagogy can enhance learners’ motivation” had the highest mean score (Mean = 3.85, SD = 0.86), and “students who learned to give and receive peer-feedback” had the least mean score (Mean = 3.72, SD = 0.86). Lastly, under emotional engagement, the level of engagement were found to be high in the items as “technology can transform the interest and feelings of students”, “technology can reduce boredom”, “technology can increase happiness”, and “students feel comfortable to participate in online discussion”, but a moderate in “technology can reduce anxiety”. But, in overall items under this dimension the statement “technology can transform the interest and feelings of students” had a highest mean score (Mean = 3.91, SD = 0.85) and “technology can reduce anxiety” had least mean score (Mean = 3.64, SD = 0.99). The significant t-value in each items showed that the level of engagement were found to be high with respect to assumed population mean.

The dimension-wise relationship is presented in Fig 3 in detail. The relation was found to be significant among all items. Additionally, the relationship was calculated based on the average of items among related dimensions which are visually presented in Fig 3. The correlation with SE was found to be significantly higher with CE (r = 0.73) whereas significant moderate relation was existed between SE with EE (r = 0.56), SE with BE (r = 0.69), CE with EE (r = 0.69), BE with CE (0.68), and BE with EE (r = 0.52) [66].

### Model fit indices with threshold criteria

The sample size was 402 and the observed variables were 19 in this study. Hence, the sample size was sufficient for SEM analysis [67]. The significant value of the chi-square was considered in the models [68] because of appropriate fit of remaining indicators as root mean square error of approximation (RMSEA) was 0.06 (<0.08), goodness-of-fit statistic (GFI) was 0.92 (>0.90), adjusted goodness-of-fit statistic (AGFI) was 0.89 (near to threshold value 0.90), standardized root mean square residual (SRMR) was 0.04 (<0.08), normed-fit index (NFI) was 0.93 (>0.09), comparative fit index (CFI) was 0.95 (>0.90), and Tucker-Lewis index (TLI) was 0.95 (>90), and incremental fit index (IFI) was 0.96 (>0.90), all of them were good fit [67–72] in Model 1 and similar acceptable results were measured in remaining models also. Some modification indices were employed to improve the threshold criteria which is presented in Table 3.

**Table 3:** Model fit indices in SEM (n = 402).

Indicators	Effect on BE (Model 1)	Effect on SE (Model 2)	Effect on CE (Model 3)	Effect on EE (Model 4)	Threshold criteria	Accepted?
CMIN	374.91	358.01	430.18	386.18		
DF	144	144	145	145		
CMIN/DF	2.60	2.49	3.00	2.66	<5	Yes
GFI	0.91	0.92	0.90	0.91	>0.90	Yes
AGFI	0.88	0.89	0.87	0.88	>0.90	Yes
NFI	0.92	0.93	0.91	0.92	>0.90	Yes
IFI	0.95	0.96	0.94	0.95	>0.90	Yes
TLI	0.94	0.95	0.93	0.94	>0.90	Yes
CFI	0.95	0.95	0.94	0.95	>0.90	Yes
RMSEA	0.06	0.06	0.07	0.06	<0.08	Yes
SRMR	0.05	0.04	0.06	0.05	<0.05	Yes

### Results based on hypothesized models

**Fig 4:**
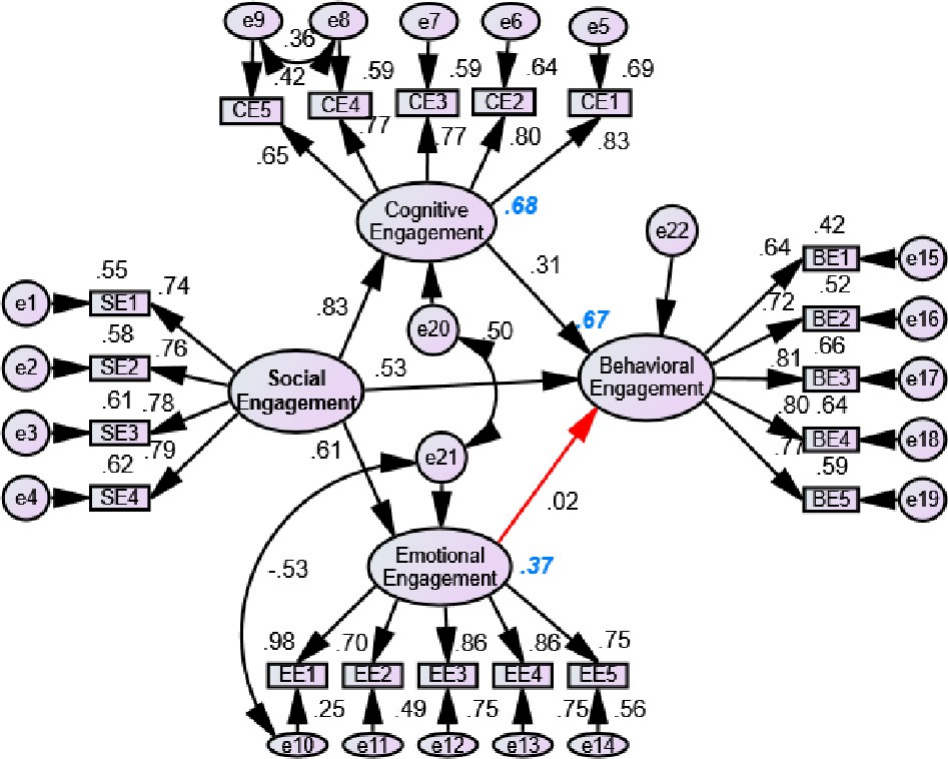
Effect of social, emotional and cognitive engagement on behavioral engagement (Model 1: Red arrow represents insignificant and black arrow represents significant results).

**Fig 5:**
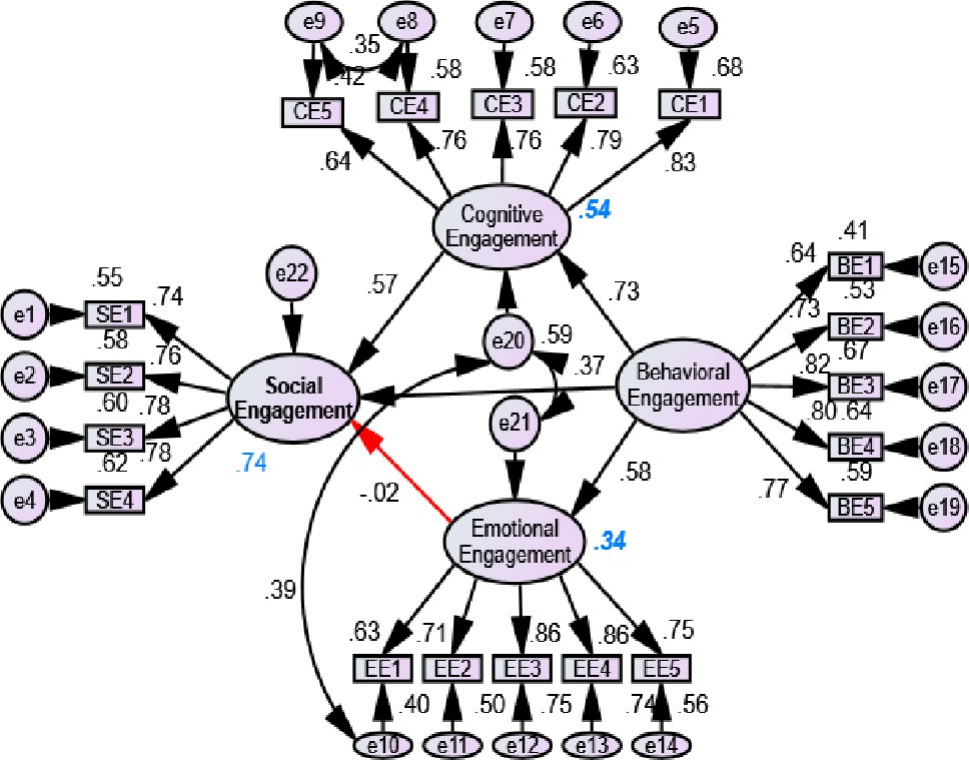
Effect of behavioral, emotional and cognitive engagement on social engagement (Model 2: Red arrow represents insignificant and black arrow represents significant results).

Fig 4 shows the result of the SEM based on different hypotheses. The model explained 68%, 67%, and 37% variance on cognitive, behavioral, and emotional engagements respectively. Social engagement had a positive significant effect on cognitive engagement with a beta value of 0.83 indicating that if SE was increased by one SD, the CE would be increased by 0.83 SD. Similarly, SE had a significant positive effect on BE (beta = 0.61) and BE (beta = 0.53). Additionally, CE had a significant effect on BE. However, the EE had no significant effect on BE. Based on lambda value, CE5, SE1, EE2, and BE1 all had the least, and CE1, SE4, EE1, and BE4 had the highest contribution to determine CE, SE, EE, and BE, respectively with compared to items in respective dimensions. Fig 5 shows the effect of BE on SE, CE and EE, similarly CE and EE on SE. The model explained 54%, 74%, and 34% in CE, SE, and EE, respectively. The BE had a positive significant effect on CE (beta = 0.73), BE (beta = 0.37), and EE (beta = 0.58) indicating that the increment of behavioral engagement enhanced the cognitive, social, and emotional engagement of learners. Similarly, CE (beta = 0.57) had a positive sign on SE. However, the EE had a negative role (beta = -0.02) to determine SE. Nonetheless, this result was still insignificant.

**Fig 6:**
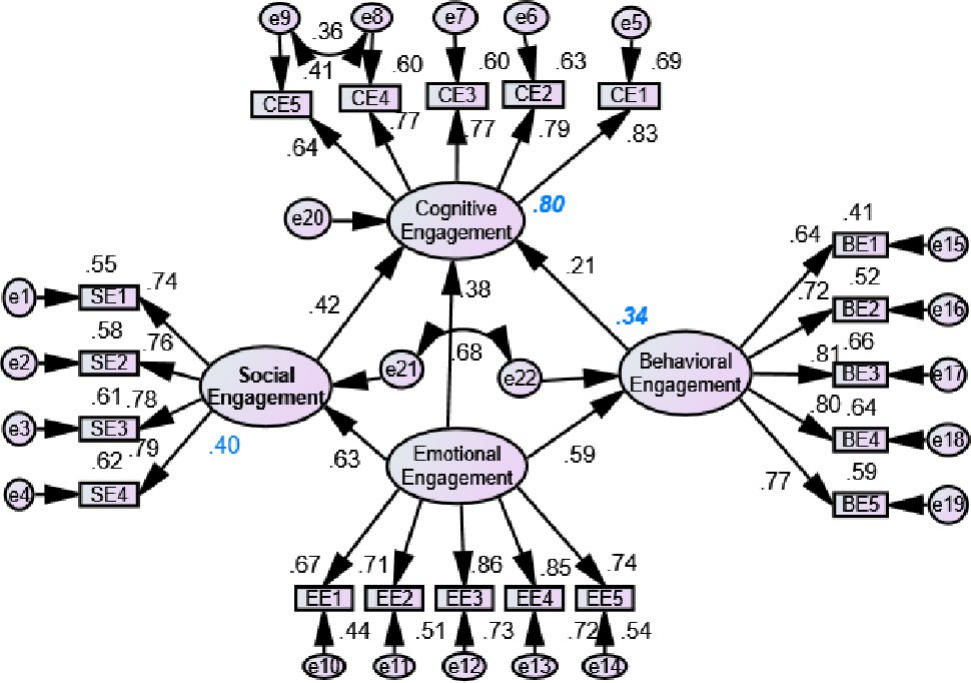
Effect of behavioral, emotional and social engagement on cognitive engagement (Model 3).

**Fig 7:**
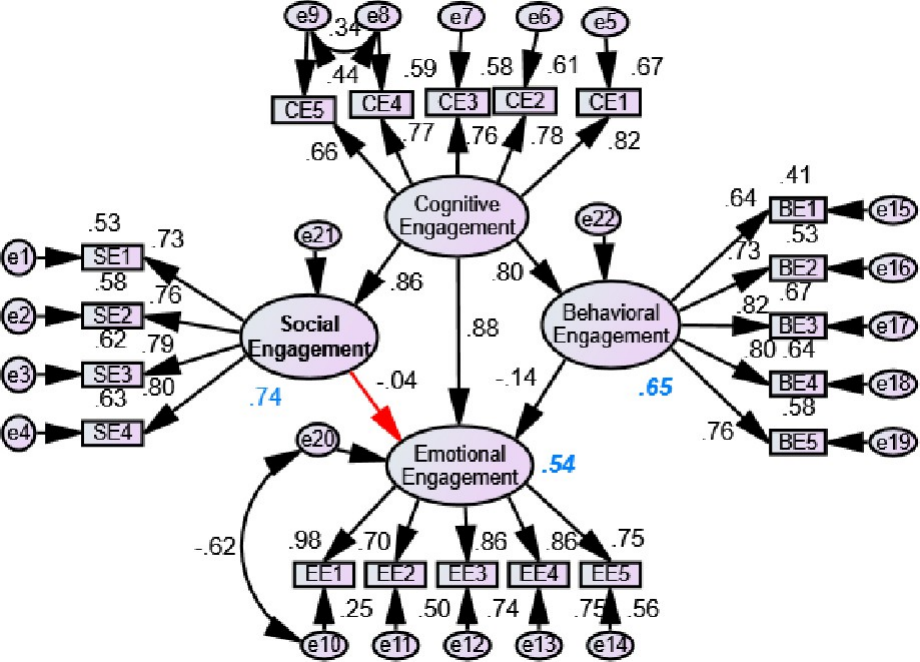
Effect of behavioral, cognitive and social engagement on emotional engagement (Model 4: Red arrow represents insignificant and black arrow represents significant results).

Cognitive engagement was considered as dependent variable in Model 3. Fig 6 shows that the SE, EE, and BE had significant positive effects on CE. The role of social engagement was found to be significantly high to determine the cognitive engagement with the highest beta value. Additionally, the emotional engagement had significant positive effect on social and behavioral engagement. Similarly, Fig 7 shows that the effect of CE and BE had significant effect on emotional engagement, whereas CE had significant effects on SE and BR. The CE was the main significant predictor to the EE, and BE had a negative contribution to determine the EE. Based on all models, the SE and BE had a high impact on CE, and CE had a high effect on BE and SE. Additionally, EE had the least insignificant effect on BE and CE, whereas SE had also same result on EE.

Table 4 shows the contribution of each item in each latent variable. The factor weight on behavioral engagement was found to be high in BE3(0.183) and BE4(0.204) indicated that when the measured variable BE3 and BE4 went up by 1 unit, the predicted value for the latent variable academic engagement would go up by 0.183 and 0.204 units, respectively. Similarly, the contribution of EE3 and EE4 had a greater role to determine EE, and CE1 and CE4 had a greater role to determine the CE, and SE3(0.178) and SE4(0.197) had a greater role to determine SE. However, none of the observed variables had a negative contribution to all measured latent variables.

**Table 4:** Factor score weights of each item with all latent variables.

Items	Behavioral engagement	Emotional engagement	Cognitive engagement	Social engagement
BE1	0.106	0.001	0.010	0.017
BE2	0.116	0.001	0.011	0.019
BE3	0.183	0.001	0.018	0.030
BE4	0.204	0.001	0.020	0.034
BE5	0.173	0.001	0.017	0.029
EE1	0.001	0.080	0.016	0.006
EE2	0.001	0.091	0.018	0.006
EE3	0.003	0.193	0.038	0.014
EE4	0.002	0.176	0.035	0.012
EE5	0.001	0.087	0.017	0.006
CE1	0.025	0.028	0.167	0.038
CE2	0.020	0.022	0.135	0.031
CE3	0.018	0.021	0.124	0.028
CE4	0.026	0.029	0.174	0.040
CE5	0.014	0.016	0.097	0.022
SE1	0.028	0.007	0.026	0.158
SE2	0.031	0.007	0.028	0.172
SE3	0.032	0.008	0.029	0.178
SE4	0.035	0.008	0.032	0.197

## Discussion

The purpose of the research was to identify the level of learner engagement in terms of behavioral, social, emotional, and cognitive activities in virtual learning of mathematics during the COVID-19 pandemic in Nepal. The level of engagement was found to be high in almost all items indicating that learners can be activated on their different activities during virtual learning, almost similar way like face-to-face classes. Additionally, these findings also suggest that the engagement of students on online modes of instruction in social, behavioral, cognitive, and emotional aspects can be transformed through the use of technology [27]. Technology can reform an internal process of education [11] with a higher level of cognitive, social, and emotional engagement of students in online classes than behavioral engagement reflects that the learners and tutors should have more focus on behavioral engagement-related activities [3]. The learning engagement on self-learning and responsible for their learning by doing found to be good which was also the beauty of virtual learning [49, 54].

The active role of learners to accomplish their homework and assignment, aware of self-evaluation, and practiced collaboration with peers were found to be moderate indicators of learner engagement. Influence of technology was found to be high indicating that the learners are active in social activities when they use social media and other learning platforms in online learning [54]. However, their engagement in academic activities was found to be comparatively low. Additionally, the level of learners’ engagement to observe community norms by using technology was moderate indicating that they might be habituated to use digital resources in nonacademic activities. Such activities with technological tools may lead to issues with ethical and moral values associated with the use of digital technology. It was found that technology can transform interest and feelings, increase happiness and reduce boredom, sadness, and anxiety indicating that technology adoption in virtual learning may have a positive impact on the emotional engagement [44].

The relationships between the behavioral, social, cognitive, and emotional engagement were found to be significant and positive indicating that increasement in any type of engagement may support in increasing the others [22]. The SEM results show that social engagement is a main contributing factor to determine the cognitive, emotional, and behavioral engagement. Additionally, cognitive engagement has a significant contribution to determining behavioral engagement [57]. Schools and universities may enhance the online learning outcome through learner enhancement in academic activities [10, 14, 16]. However, the finding of this research suggest that social and cognitive engagement have a significant role to determine behavioral engagement. In this context, the results showed that the values related to emotional and academic engagement should be enhanced to promote learners’ academic performance in mathematics (and other disciplines). Students’ behavioral engagement is the main predictor to determine the cognitive, emotional, and social engagement of learners. Therefore, the engagement of learners on a self-learning, accomplishing the homework and assignment, self-evaluation, collaboration for learning, and making them responsible for learning by doing should be considered in online learning of mathematics [49]. Additionally, emotional engagement has a significant role to determine social engagement. The learners should be encouraged in self-directed learning, peer-evaluation, and transforming their role as content experts, and collaboration, and motivation towards digital pedagogy. The emotional engagement of students also enhances motivation [22] and reduces attrition rates [24]. Because of having several digital technology-related issues, such as access to the Internet, digital devices, technical support, digitally trained human resources, and hardware in the developing countries, like Nepal, students may have less motivation towards virtual learning. Nevertheless, virtual learning has been an inevitable component for the continuation of students’ learning after the outbreak of COVID-19 pandemic in Nepal. Hence, effective and meaningful engagement of students in a virtual learning environment has been an issue in the Nepalese context.

### Implications

The results of this study may have implications for understanding the status of learners’ engagement in different activities during virtual learning in mathematics. The findings also provided a better understanding of how learners can be engaged in academic activities while learning mathematics online. More importantly, the findings demonstrate the importance of learners’ engagement in mathematics learning during the period when physical classes are not possible to run. This study demonstrated the potential framework for learners’ engagement. Applications foreseen are not only in the area of mathematics learning but also in learning other subjects. The results of this research are important for all the students, teachers, administrators, and other educators to make appropriate policies for learners’ engagement during virtual learning with the selection of appropriate instructional design in virtual learning.

The findings of the study have both pedagogical and policy implications. In terms of pedagogy, social, emotional, and behavioral engagements have significant contributions to the cognitive engagement which may lead to improved learning outcomes. Such engagements are interconnected and mutually inclusive to influence students’ creativity, critical thinking, and collaboration [73]. Hence, concerned stakeholders should focus on enhancing learners’ engagement in self-learning, accomplishing assignments on time, being aware of self-evaluation, collaboration, participation in social activities, forming study groups, reducing boredom and anxiety, and increasing happiness to promote a greater motivation towards learning. If students are distanced and not engaged in online learning groups, collaboration will be weak [74]. For these changes, they should have access to self-learning materials, peer feedback, and collaboration for completing assignments. Additionally, the emotional engagement has the highest effect on the remaining engagement dimensions. Hence, transforming the interests of learners, reducing their anxieties and boredoms, increasing happiness and managing comfortable environment for the students to participate in online discussion should be priorities of schools and teachers.

The policy implication of this study is that the findings may guide policy makers, in education in general and mathematics education in particular, to emphasize learners’ active engagement in tasks, activities, and collaboration through resource support and technological infrastructures. The findings suggest that the four kinds of engagement: social, behavioral, cognitive, and emotional engagement, all interact with and influence each other in a powerful way. Therefore, the curriculum, textbooks, teaching and learning, and assessment practices in mathematics through an online learning environment can be considered as a complement (but not a full replacement) to face-to-face learning mode to enhance students’ achievement with positive perceptions through relevance, collaboration, opportunity, quality, and development [75, 76].

## Conclusion, limitation, and recommendation

Findings of this study indicated that learners’ engagement was found to be high in social, emotional, and cognitive dimensions during online learning in Nepal. Social and cognitive engagement are the main contributing factors in determining behavior engagement. Whereas social engagement has a great role in determining cognitive engagement. Similarly, behavioral and cognitive engagement were important predictors of social engagement.

The study has several limitations in terms of samples and models to study four kinds of engagement. The engagement of learners was measured by the mathematics teachers’ self-reports. As a sample, only selected teachers were considered as the population, and the study did not cover all geographical locations. The study was also limited to survey design. The socio-demographic and digital technological backgrounds were not included in the study. Further research should seek to address these issues by taking students as a sample, comparing results based on different socio-demographic characteristics, and using other research designs by taking different geographical areas of the nation as a sample.

Based on the findings of the study, we would like to recommend that schools and mathematics teachers together with policymakers in education should look upon students’ engagement as a complex construct where social, behavioral, cognitive, and emotional engagement affect each other and students’ learning. Hence, it is recommended that students’ engagement be considered a major factor that influences their learning and achievement in mathematics and other disciplines and that they focus on creating a school environment for both face-to-face and virtual learning. There is a need for further studies to promote students’ engagement in meaningful learning with creativity, critical thinking, collaboration, and effective communication skills for the twenty-first century to achieve the sustainable development goals through effective means of education in Nepal and elsewhere.

## Supporting information

S1 Appendix (DOCX)

S1 Data (SAV)
