# Supplementary material for: Behavioral, cognitive, emotional and social engagement in mathematics learning during COVID-19 pandemic
Source: PLoS One. 2022 Nov 22;17(11):e0278052. doi: 10.1371/journal.pone.0278052 (PMC9681528; doi:10.1371/journal.pone.0278052)
Supplement: S1 Appendix — (DOCX) [file pone.0278052.s001.docx]

**Appendix 1**

**Transformation on Learners' Engagement in Mathematics Learning During COVID-19 Pandemic**

Group A

Personal background

Name:

Gender:

Qualification:

Teaching level:

Group B

Engagement of Learners in Mathematics Learning During COVID-19 Pandemic

Based on your teaching experience as compare to face-to-face class with virtual classes during pandemic, please tick on one and only one options in each question (SD-strongly disagree, D-disagree, N-neutral, A-Agree, SA-strongly agree)

| Items with categories | SD | D | N | A | SA |
| --- | --- | --- | --- | --- | --- |
| **Behavioral Engagement** |  |  |  |  |  |
| The student seemed engaged in self-learning |  |  |  |  |  |
| Students are more active to accomplish the homework and assignment on time |  |  |  |  |  |
| Students are becoming more aware of self-evaluation |  |  |  |  |  |
| Students have practiced collaboration with peers on academic discussion |  |  |  |  |  |
| Students are responsible and focused during the online classes |  |  |  |  |  |
| **Social Engagement** |  |  |  |  |  |
| Students can observe community norms by using technology |  |  |  |  |  |
| Students participate in different social activities through technology |  |  |  |  |  |
| Students are active in forming a study group in the online forum and take participate in group work |  |  |  |  |  |
| Students can learn ways of working in a group through technology |  |  |  |  |  |
| **Cognitive Engagement** |  |  |  |  |  |
| Online learning enhances learner's motivation towards learning |  |  |  |  |  |
| Online pedagogy has encouraged learners to manage self-learning materials |  |  |  |  |  |
| Technology can develop/transform students as self-directed learner |  |  |  |  |  |
| Students learned to give and receive peer feedback and correct their own mistakes |  |  |  |  |  |
| Students can collaborate with peers in doing homework and assignment |  |  |  |  |  |
| **Emotional Engagement** |  |  |  |  |  |
| Technology can transform the interest and feelings of students |  |  |  |  |  |
| Technology can reduce boredom |  |  |  |  |  |
| Technology can increase happiness |  |  |  |  |  |
| Students feel comfortable participating in online discussion |  |  |  |  |  |
| Technology can reduce anxiety |  |  |  |  |  |
